# A survey of the frequency of cytomegalovirus-associated diarrhea in immunocompromised patients using a non-invasive method

**Published:** 2018-04

**Authors:** Mahmoud Agholi, Akbar Safaei, Mani Ramzi, Gholam Reza Hatam, Jamal Sarvari

**Affiliations:** 1 HIV/AIDS Research Center, Fasa University of Medical Sciences, Fasa, Iran; 2 Department of Parasitology and Mycology, Fasa University of Medical Sciences, Fasa, Iran; 3 Department of Pathology, School of Medicine, Shiraz University of Medical Sciences, Shiraz, Iran; 4 Hematology Research Center, Shiraz University of Medical Sciences, Shiraz, Iran; 5 Department of Parasitology and Mycology, School of Medicine, Shiraz University of Medical Sciences, Shiraz, Iran; 6 Basic Sciences in Infectious Diseases Research Center, ShirazUniversity of Medical Sciences, Shiraz, Iran; 7 Department of Bacteriology and Virology, School of Medicine, Shiraz University of Medical Sciences, Shiraz, Iran; 8 Gastroenterohepatology Research Center, Shiraz University of Medical Sciences, Shiraz, Iran

**Keywords:** *Cytomegalovirus*, Stool, Immunocompromised, Diarrhea, PCR

## Abstract

**Background and Objectives:**

*Cytomegalovirus (CMV)* infection is the most common viral opportunistic infection causing gastrointestinal diseases such diarrhea and colitis in immunocompromised patients. The development and performance of a robust and sensitive PCR assay are usually evaluated to detect CMV DNA in human fecal specimens. In this study, our aim was to detect CMV DNA in stool samples taken from patients with HIV/AIDS, cancer, and transplant recipient patients with chronic and persistent diarrhea using a non-invasive method.

**Materials and Methods:**

A total of 633 immunocompromised patients (451 males and 182 females) suffering from persistent or chronic diarrhea were included in this study. Among them, 392 were HIV/AIDS patients, 151 had cancer and were receiving chemotherapy, and 90 were recipients of a solid organ or bone marrow transplant. CMV genome was extracted from the stool samples using phenol: chloroform: isoamyl alcohol method. CMV DNA was identified by polymerase chain reaction using sequence specific primers on genomic DNA.

**Results:**

Looking at the frequency of CMV DNA in 392 HIV/AIDS patients, we found that only 5 patients (1.27%) were positive for CMV genome, while this frequency was 4.63% (7/151) and 5.5% (5/90) in patients with cancer receiving chemotherapy and in those with solid organ or bone marrow transplant, respectively.

**Conclusion:**

The results of this study revealed that the cause of chronic or persistent diarrhea in HIV/AIDS, cancer, and graft recipient patients might be related to CMV infection. Accordingly, we recommend a non-invasive method, such as stool sample, as a first line of diagnosis of enteritis when the physician suspects that a patient has CMV infection.

## INTRODUCTION

The immunocompromised hosts are mainly HIV/AIDS patients, those receiving immunosuppressants for cancer therapy, or organ transplantation. Opportunistic pathogens do not generally cause any disease in healthy individuals, but they can cause significant occasional deadly diseases in a susceptible host (1, 2). Numerous opportunistic pathogens, including human cytomegalovirus (HCMV), might be associated with diarrhea. HCMV, as a large enveloped herpes virus, is a persistent virus with a global distribution. CMV prevalence varies considerably, according to geographical areas, age, and socioeconomic status, ranging from 30% to 100% (3). Primary infection in children is almost asymptomatic although symptomatic cases may occur as an infectious mononucleosis-like syndrome mainly in adults (3). However, the virus can exert its full potential when the immune system compromises and can even cause life-threatening infection, as documented in immunosuppressed patients. Moreover, the role of CMV infection in inflammatory situations (4) and certain cancers has been recently acknowledged by several studies (5). CMV is highly important among opportunistic viral agents in immunosuppressed situations that may occur either as a result of reactivation of a hidden virus or as a primary infection (4, 5). Colitis, retinitis, and esophagitis are the most common complications of CMV infection, mostly occurring in immunocompromised patients, including those with congenital or acquired immunodeficiency disease (6).

CMV infection is the most common infectious complication following transplantation and occurs in 60% to 70% of organ transplant recipients (7). Infection with gastrointestinal (GI) involvement, including colitis, occurs in 10% to 30% of organ transplant recipients (8) with such symptoms as abdominal pain, anorexia, malaise, nausea/vomiting, diarrhea, and bleeding. Most of the manifestations are usually secondary to separate erosions or ulcerations that may lead to life-threatening complications, including GI damage and massive bleeding (9). Virus isolation or detection of virus antigens in the body fluids or tissue specimens is defined as CMV infection (10). CMV also infects the epithelial and mesenchymal cells, destroys the cells, and causes ulcers on the epithelial layers of different organs, such as intestine and colon (8). For patients, diarrhea is bothering and in convenient; moreover, prolonged diarrhea could lead to dehydration, weight loss, increased serum creatinine, and fluctuating level of immunosuppressive drugs (11). Chronic diarrhea has also been documented as the main complication of HIV infection and the acquired immunodeficiency syndrome (AIDS). This characteristic in HIV/AIDS is very important, as the disease has been referred to as a ‘slim disease’ in Africa as a consequence of the combination of chronic diarrhea and weight loss (12).

Although studies looking at the seroprevalence of CMV in healthy population in Iran is scanty, few studies have reported the high prevalence of CMV antibody in some high-risk groups such as those with coronary artery disease (83.2%) (13), HIV positive individuals (94%) (14), and pregnant women (90%–97%) (15, 16). The non-specific clinical symptoms of intestinal CMV disease, including diarrhea and weight loss, do not permit differential diagnosis of other intestinal pathogens in most cases (8, 17). Early etiologic diagnosis is important for both the management of therapy and patients (18). There are several diagnostic tools for CMV colitis including serologic analysis, viral cultures, genome detection using polymerase chain reaction (PCR), imaging studies, and histological review of endoscopic or surgical biopsy specimens. However, the gold standard for diagnosis is identifying CMV inclusion bodies at the affected sites. Considering the high prevalence of IgG anti-CMV antibodies in the general population, IgM assay is not very helpful in diagnosing CMV colitis. While the presence of IgM anti-CMV antibodies corresponds to the current CMV infection, their presence does not establish the diagnosis of tissue-invasive disease. Furthermore, more than 10% of patients with active CMV infection are usually negative for IgM antibody (8). CMV cell culture is not often helpful from clinical point of view, as it requires several weeks to get the results. Moreover, CMV stool cultures are positive in approximately 30% of patients with documented CMV colitis (8).

During active CMV infection, virus shedding occurs in urine, respiratory secretions, and stool of infected patients (19, 20). Qualitative CMV DNA from stool by PCR is a relatively fast and non-invasive technique with very high sensitivity (8, 20, 21), which may offer the possibility to test the concurrent CMV infections in patients with chronic diarrhea without the need for obtaining endoscopic biopsies (22). Some studies have suggested a high accuracy of this non-invasive testing method to detect CMV DNA in the stool samples as compared to mucosal biopsies (22, 23). In this regard, in a study conducted by Ganzenmueller et al., the sensitivity and specificity of stool sample in diagnosing CMV intestinal disease were found to be 67% and 96%, respectively, and have been shown to be more sensitive and specific than the histopathology and immunohistochemistry as a gold standard method (23). In line with these findings, molecular assays, such as PCR, on stool samples might serve as a useful tool for diagnosing CMV gastrointestinal infection. Unfortunately, less data are available on intestinal CMV infection in immunosuppressed patients in Iran. The present study aimed at detecting CMV DNA in stool samples collected from patients with HIV/AIDS, cancer, and graft recipients with chronic and persistent diarrhea.

## MATERIALS AND METHODS

### Samples.

A total of 633 patients, including 392 HIV/AIDS patients, 151 patients with cancer who were receiving chemotherapy, and 90 solid organ or bone marrow transplant recipients suffering from recurrent chronic or persistent diarrhea, were included in this study from September 2009 to June 2016 (Table 1). Samples were collected from 2 teaching hospitals in affiliation with Shiraz University of Medical Sciences. A structured questionnaire was used to collect sociodemographic characteristics, potential risk factors, and clinical findings. Written informed consent was obtained from each participant, and the study was approved by the local Ethics Committee of Shiraz University of Medical Sciences. Fecal specimens were collected and immediately transported to the laboratory. The fecal specimens were stored at −20°C prior to DNA extraction.

**Table 1:** Incidence rate of *Cytomegalovirus* detected from diarrheic immunodeficient patients (n=633)

**Patients’ Samples (%)**	**No. of examined samples**	**No. of positive**
HIV/AIDS 392 5 (1.27%) Solid organ or bone marrowtransplantrecipients	90	5 (5.5%)
Patients with cancer receiving chemotherapy	151	7 (4.63%)
Total	633	17 (2.68%)

### DNA extraction.

DNA extraction was performed on 200 mL of each fecal sample using the phenol/chloroform/isoamyl alcohol method. Briefly, an equal volume of lysis buffer containing 10 mMTris/HCl at pH 8, 0.1 M EDTA at pH 8, 0.5% SDS, and 200 μg/mL proteinase K was added to the suspension. The resultant mixture was placed in a dry block at 55°C for 3 hours and then heated at 95°C for 10 minutes to inactivate proteinase K. Supernatant was obtained after centrifugation, and proteinase K digestion was subjected to DNA extraction, once with equal volume of phenol/chloroform and once with phenol/chloroform/isoamyl alcohol (25:24:1). DNA was precipitated with double volume of cold absolute ethanol and sodium acetate (3 mM) for 1 hour at −20°C, followed by centrifugation at 14000 rpm for 10 min at 4°C (1, 2). The DNA pellet was then washed with 70% ethanol, dried, and resuspended in 30 μL sterile distilled water. The quality and quantity of DNA were finally determined spectrophotometrically at 260 and 280 nm.

### Detection of CMV DNA by PCR.

**Fig. 1:**
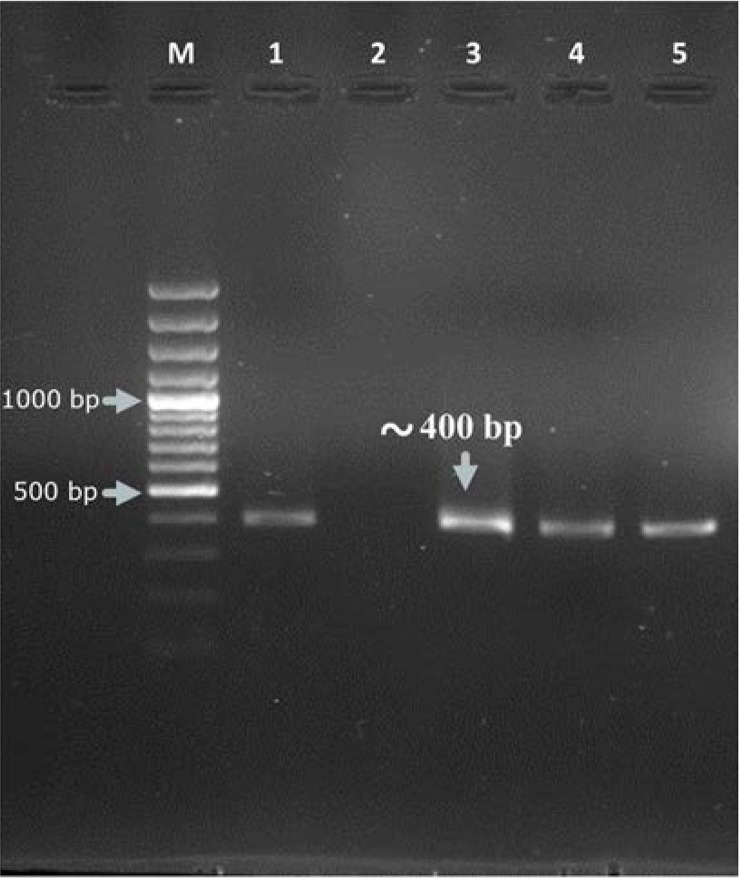
Electrophoresis of PCR products of cytomegalovirus on 1.5% agarose gel. Lane M: 100 bp DNA size marker. Lanes 1 and 2 indicate positive and negative controls, respectively. Lanes 3 to 5 indicate CMV positive samples.

CMV DNA was amplified using a primer pair selected from previously described oligonucleotides from the *Hind*III–X fragment region (F: 5′- GGA TCC GCA TGG CAT TCA CGT ATG T3′ and R: 5′-GAA TTC AGT GGA TAA CCT GCG GCG A -3′ [406 bp] (24). One microgram of DNA in 5 μL of water was added to 45 μL of reaction mixture containing 20 mM Tris-HCL hydrochloride (pH 8.3), 60 mM KCl, 2 mM MgCl_2_, 200 mM (each) deoxynucleoside triphosphates (Pharmacia, Uppsala, Sweden), 25 pmol of each primer, and 1.5 U of thermostable Taq DNA polymerase (Cinagene, Iran). PCR amplification was done in a thermocycler using the following cycling programs: 1 cycle of 95°C for 5 min, 35 cycles of 94°C for 1 min, 55°C for 2 min, 72°C for 3 min, and 1 cycle of 72°C for 10 min. The PCR products were then analyzed by 2% agarose gel electrophoresis, and the presence of a 406 base pair amplicon was considered as CMV DNA (Fig. 1).

### Statistical analysis.

Statistical analysis was performed using SPSS version 16 and EPI inform software. P-values <0.05 were considered statistically significant in two-sided tests.

## RESULTS

A total of 633 diarrheic patients (rages: 3 months to 94 years) were enrolled in this study, among whom 451 were male. Out of 392 HIV/AIDS patients, 303 were male and 89 female; in the cancer group, 97 patients were male and 54 female; and in the transplant recipient group, 51 patients were male and 39 female. Results obtained from PCR revealed that only 2.68% (17 out of 633) of the samples were positive for CMV DNA. As demonstrated in Table 1, 1.27% (5/392), 4.63% (7/151), and 5.5% (5/90) of the samples were found to be positive for CMV genome in HIV/AIDS patients, in those with cancer undergoing chemotherapy, and in organ transplant recipients, respectively. Analyzing the frequency of CMV positive samples among the 3 groups showed no statistically significant association. In HIV/AIDS group, all positive samples for CMV genome were only found in those with CD4 below 100 cells/mm^3^ and, interestingly, all patients with CD4 higher than 100 cells/mm^3^ were negative (Table 2). With regards to gender in CMV positive patients (n= 17), 11 were male and 6 female. In the HIV/AIDS group, 4 positive samples belonged to males and 1 to a female. In the solid organ or bone marrow transplant recipients, 3 positive samples belonged to males and the rest to females. In patients with cancer receiving chemotherapy, 4 positive samples belonged to males and 3 to females. Statistical analysis showed no association between CMV DNA positivity and the gender of the participants of the 3 groups.

**Table 2:** *Cytomegalovirus* detected as a causative agent of diarrhea in HIV/AIDS patients

	**Cd4<200 cells/mm^3^ (n=224)**	**Cd4 200–500 cells/mm^3^ (n=117)**	**Cd4>500 cells/mm^3^ (n=51)**	**Total (392)**
*Cytomegalovirusa*	5	0	0	5
Total	5/224 (2.23%)	0/117 (0%)	0/51 (0%)	5/392 (1.27%)

a Detected using *Hind*III–X fragment region-specific primers

## DISCUSSION

The existence of CMV DNA in fecal samples of immunocompromised patients by PCR has been described, suggesting that the presence of CMV DNA in feces might be useful as a non-invasive method to detect CMV-associated intestinal disease, particularly in high-risk groups (20, 21). Furthermore, detecting CMV DNA in the fecal sample may help screen the high-risk patients who suffer from colon disease and are suspected to have CMV infection (20). CMV colitis mostly occurs in immunocompromised patients, including those with acquired or congenital immunodeficiency syndrome, those undergoing immunosuppressive therapy, and solid organ transplant recipients (6). Over the last 3decades, major advances in the management of CMV infection in immunocompromised patients have been accomplished through the invention of new diagnostic methods and antivirals. In addition to the direct effects resulting from CMV infection, CMV-associated indirect effects, such as increased risk of acute or chronic allograft rejection and other inflammatory situations, have gained much attention (3). The clinical symptoms of gastrointestinal CMV disease, including diarrhea and weight loss, are non-specific and do not allow a specific diagnosis to be made for many cases; therefore, using laboratory methods, such as molecular techniques, is necessary for differential diagnoses in these settings (20).

Chronic diarrhea has been recognized as a major complication of HIV/AIDS since the early days of the pandemic. Case series from different countries indicate that around 40% to 80% of HIV/AIDS patients will experience diarrhea (25) that together with weight loss can cause serious problems for these patients (25). After examining 392 stool samples collected from HIV/AIDS patients, we found that only 5 were positive for CMV genome in PCR. In a study conducted by Michel et al., out of the samples of 16 HIV patients with chronic diarrhea, 5 and 4 samples were positive in histological and PCR investigations, respectively (20). In the present study, the frequency of CMV DNA observed in the HIV/AIDS patients’ stool samples was 1.27%. In this regard, Nahar et al. reported 12.3% (37 out of 300) GI CMV infection in patients with HIV/AIDS and in those who were treated with immunosuppressive drugs with ulcerative colitis (26). The low frequency of CMV DNA in our study might be associated with different assay methods, the stage of HIV/AIDS target group concerning the CD4 count, and sample type, as the stool samples of other studies have been collected from patients with colitis; however, in our study, the stool samples were taken from patients with only chronic diarrhea but not colitis.

HIV targets and induces the apoptosis of CD4+ T lymphocytes and results in progressive decline of these cells. The risk of CMV disease in HIV-infected patients is increased as the CD4 count falls. Decrease in CD4+ T cells can cause progressive immunosuppression and increase the risk of CMV-related diseases more rapidly (27). Monitoring this decline in plasma is an important marker of immune status and a key tool in guiding the physician to initiate ART and determine the type of opportunistic infections such as CMV colitis (25). According to the CD4 count, all our positive samples for CMV DNA had CD4 less than 100 cells/mm^3^, which correlate with severe immune system suppression.

CMV infection occurs in most of transplanted patients and is one of the most common viral complications in these patients (11). Although most CMV infections are mild and transient, some symptoms are severe and can be life-threatening (28). The incidence of CMV gastric infection is unusually high, affecting a considerable number of patients, particularly during the first 6 to 12 months after organ transplantation (28, 29). Based on our results, 5.5% stool samples collected from solid organ transplant recipients were positive for CMV DNA. Although Sakr et al. (30) and Ginsburg et al. (28) reported the incidence of diarrhea in transplanted populations to be 10% to 43%, other available data suggest that the overall incidence of post-liver transplant gastrointestinal CMV is around 1% to 2% (28).

Studies looking at the frequency of GI CMV infection in transplant recipients have yielded inconsistent results. Indeed, similar to our findings, in a study performed by Burak et al. (31), only 2% (2/93) of liver transplant recipients reported to be positive for GI CMV infection. Unlike our results, a relatively high frequency of GI CMV infection in transplant patients was reported by several groups. In this regard, the frequency of GI CMV infection in bone marrow (20), solid organ recipients (32), and liver transplant recipients (33) have been reported to be 17.6% (3/17), 13% (6/52), and 10% (3/31), respectively. Moreover, in a study conducted by Maes et al., 7% (8/108) of renal transplant patients with severe diarrhea were reported to be positive for CMV in PCR (11). Applying different tests with different sensitivities to detect CMV infection and the severity of immunosuppression might be behind the conflicting results reported from various studies. Furthermore, researches revealed some case reports of CMV-associated diarrhea in solid organ transplant recipients. In this regard, Merrikhi et al. reported CMV colitis in a 10-year-old girl after kidney transplantation (10). CMV enteritis after autologous peripheral blood stem cell transplantation has been reported by Kozuka et al. (34).

In our study, 4.63% (7/151) of the patients with cancer who were receiving chemotherapy were positive for CMV DNA in their stool diarrheal samples. In line with our findings, Nomura et al. reported severe CMV enterocolitis after standard chemotherapy in a patient with non-Hodgkin’s lymphoma (35). Moreover, Van den Brande et al. (36) and Kitahara et al. (37) reported CMV colitis during chemotherapy in a locally advanced hypopharyngeal cancer and esophageal cancer, respectively. Oshima et al. have also reported the successful treatment of a 84-year-old patient with adult T-cell leukemia lymphoma with ganciclovir (38).

Although CMV infection of the gastrointestinal tract has been commonly recognized in immuno-compromised patients, it might infrequently be seen in immunocompetent individuals, which could be detected in stool samples using PCR (26, 39, 40).

According to our and others’ findings, positive fecal PCR results are diagnostically valuable and might help avoid invasive diagnostic procedures, such as endoscopy, but negative CMV PCR results from fecal samples cannot exclude CMV intestinal disease and, therefore, need to be confirmed by alternative procedures such as analyzing intestinal biopsies.

Although the prevalence of CMV infection in solid organ/bone marrow transplant recipients was higher than those with cancer receiving chemotherapy (5.5% versus 4.63%) and HIV/AIDS patients (5.5% versus 1.27%), the difference was not statistically significant, suggesting that the type of immunosuppression might not influence the frequency of CMV infection. While the frequency of CMV was higher in females than in males, it was not statistically significant. Similar to our results, CMV infection was found to be more frequent in female patients than in the male (8/10 vs. 23/53) although the difference did not reach statistical significance (41).

In conclusion, our results indicated that chronic diarrhea that was observed in patients with HIV/AIDS, in organ transplant recipients, and in patients with cancer receiving chemotherapy might have been associated with CMV infection. Our data also support applying non-invasive and cost-benefit techniques, such as PCR, on stool samples to identify colitis-related CMV infection.
